# Phosphorylation on Syk Y342 is important for both ITAM and hemITAM signaling in platelets

**DOI:** 10.1016/j.jbc.2022.102189

**Published:** 2022-06-24

**Authors:** John C. Kostyak, Benjamin Mauri, Carol Dangelmaier, Hymavathi Reddy Vari, Akruti Patel, Monica Wright, Haritha Reddy, Alexander Y. Tsygankov, Satya P. Kunapuli

**Affiliations:** Sol Sherry Thrombosis Research Center and Department of Cardiovascular Sciences, Lewis Katz School of Medicine, Temple University, Philadelphia, Pennsylvania, USA

**Keywords:** platelet, hemostasis, tyrosine, thrombosis, spleen tyrosine kinase, CLEC-2, C-type lectin-like receptor-2, CRP, collagen-related peptide, GPVI, glycoprotein VI, ITAM, immune tyrosine activation motif, LAT, linker for activation of T-cell, PLCγ2, phospholipase Cγ2, SFK, Src family kinase, Syk, spleen tyrosine kinase

## Abstract

Immune cells express receptors bearing an immune tyrosine activation motif (ITAM) containing two YXXL motifs or hemITAMs containing only one YXXL motif. Phosphorylation of the ITAM/hemITAM is mediated by Src family kinases allowing for the binding and activation of spleen tyrosine kinase (Syk). It is believed that Syk must be phosphorylated on tyrosine residues for activation, and Tyr342, а conserved tyrosine in the interdomain B region, has been shown to be critical for regulating Syk in FcεR1-activated mast cells. Syk is a key mediator of signaling pathways downstream of several platelet pathways including the ITAM bearing glycoprotein VI (GPVI)/Fc receptor gamma chain collagen receptor and the hemITAM containing C-type lectin-like receptor-2 (CLEC-2). Since platelet activation is a crucial step in both hemostasis and thrombosis, we evaluated the importance of Syk Y342 in these processes by producing an Syk Y342F knock-in mouse. When using a CLEC-2 antibody as an agonist, reduced aggregation and secretion were observed in Syk Y342F mouse platelets when compared with control mouse platelets. Platelet reactivity was also reduced in response to the GPVI agonist collagen-related peptide. Signaling initiated by either GPVI or CLEC-2 was also greatly inhibited, including Syk Y519/520 phosphorylation. Hemostasis, as measured by tail bleeding time, was not altered in Syk Y342F mice, but thrombus formation in response to FeCl_3_ injury was prolonged in Syk Y342F mice. These data demonstrate that phosphorylation of Y342 on Syk following stimulation of either GPVI or CLEC-2 receptors is important for the ability of Syk to transduce a signal.

Immune cells express several receptors that, upon activation, their cytoplasmic tails are phosphorylated on tyrosines in a stretch of domain known as immune tyrosine activation motif (ITAM) containing two YXX(L/I) motifs (1, 2). Receptors that contain the ITAM motif include the T-cell receptor on T lymphocytes, Fc receptor subtypes on several blood cells, and the glycoprotein VI (GPVI) receptor on platelets (1, 2). There also exists a different group of receptors that contain only one YXX(L/I) motif in the cytoplasmic domain that is known as hemITAM (1, 2). The hemITAM-containing receptors include the C-type lectin-like receptor-2 (CLEC-2) on platelets and immune cells as well as dectin in macrophages (1, 2). Both ITAM- and hemITAM-containing receptors cause cellular stimulation through activation of spleen tyrosine kinase (Syk) (or its homolog ZAP-70 in T lymphocytes and NK cells) (1, 2). While it has been known that Syk can bind to phosphorylated ITAM or hemITAM (3, 4, 5), the mechanisms subsequent to its binding leading to its activation are not clear. Syk contains several tyrosine residues that are known to be phosphorylated upon its binding to the phosphorylated ITAM or hemITAM (6, 7). However, the individual roles of these tyrosine residues and their relative contributions to the function of Syk are not clear. Moreover, differential roles of Syk regulatory tyrosine residues in ITAM- and hemITAM-mediated stimulation have never been investigated. Finally, the mechanisms of tyrosine phosphorylation–dependent regulation of Syk activity in platelets remain poorly understood (8). Considering that platelets express both ITAM-containing and hemITAM-containing receptors and Syk plays a key role in platelet signaling and activation (9, 10, 11, 12, 13), we chose these cells as a model system to evaluate the role of tyrosines on Syk in its activation.

Platelets are anucleate cells that are produced by megakaryocytes (14). Their primary function is to respond to vascular damage and mediate hemostasis (15, 16). Platelets express several cell surface receptors that are necessary for these processes (15, 16). Signaling originating from a cell surface receptor causes platelet shape change, secretion of granular contents, and production of thromboxane A2, which reinforces the original signal and recruits other platelets (15, 16). Understanding how these cell surface receptors signal intracellularly is crucial to understanding the hemostatic and thrombotic process.

There are two main categories of receptors on the platelet surface: the G protein–coupled receptors and those that signal through a nonreceptor tyrosine kinase. Those that signal through a nonreceptor tyrosine kinase include the ITAM-bearing GPVI receptor and the Fc receptor for immunoglobulin, FcγRIIA, as well as the hemITAM-bearing receptor CLEC-2. Ligand engagement of these receptors results in Src family kinase (SFK) phosphorylation of tyrosine residues within the cytoplasmic domain of the receptor (10, 17, 18).

GPVI is a platelet receptor for collagen and is constitutively bound to the Fc receptor gamma chain (19). Collagen engagement of GPVI results in SFK phosphorylation of the ITAM, to which Syk binds *via* its two SH2 domains (20, 21, 22, 23, 24). Syk is then phosphorylated by SFKs and subsequently by autophosphorylation. Although the identities of tyrosine sites that are phosphorylated are established, their roles in Syk activation are not yet clear. Activation of Syk in this manner allows phosphorylation of linker for activation of T-cell (LAT) and subsequent activation of phosphoinositide 3-kinase. Subsequently, Bruton’s tyrosine kinase is activated and recruited to the signaling complex and in cooperation with LAT phosphorylates phospholipase C γ2 (PLCγ2) (25, 26). Src homology 2–containing leukocyte protein 76 (SLP76) is also phosphorylated as part of the LAT signalosome. Many of the proteins important for GPVI-mediated signaling are also important for CLEC-2-mediated signaling.

CLEC-2 is the receptor for podoplanin and is highly expressed on platelets. Engagement of CLEC-2 by podoplanin is essential for separation of blood and lymph (27, 28). Ligand binding to CLEC-2 causes tyrosine phosphorylation of the hemITAM and subsequent docking of Syk *via* its SH2 domains as well as activation of PI3 kinase and Tec kinases that phosphorylate Syk (29, 30). This elicits a downstream signaling cascade that results in platelet functional responses. While many similarities between GPVI and CLEC-2 are present, signaling events leading to Syk phosphorylation are not identical between the two receptors (29). However, in either case, Syk is required.

Syk is phosphorylated on several tyrosine residues in response to GPVI or CLEC-2 engagement. We previously showed that several tyrosine residues on Syk are phosphorylated in response to the GPVI agonist convulxin (8, 31). Furthermore, we and others have demonstrated that both Syk Y519/520 and Y346 are phosphorylated downstream of CLEC-2 (32, 33). Syk Y519/520 is located in the activation loop of Syk and appears to be crucial for Syk signaling (34, 35, 36). The molecular basis of this involvement remains not fully understood, since Syk YY519/520FF reduces Syk *in vitro* kinase activity modestly, while substantially diminishing tyrosine phosphorylation of Syk and cellular proteins, although the degree of this effect differs for various proteins (37, 38).

Studies in several cell types indicated that Y342 and Y346, located in the interdomain B (linker) region, are involved in the regulation of Syk activity and functions (8, 39, 40, 41, 42, 43). The molecular basis of their functions is not fully understood, but it appears likely that phosphorylation of these residues is involved in the transition of Syk from its autoinhibited to its active conformation (44). Although some data argue that Y342 is the most important of the regulatory tyrosine residues in the interdomain B (40, 41, 42, 43), the results vary in different cell types and for different responses, and very little data have been obtained in platelets, so we were compelled to address this problem. To begin addressing it, we produced Syk Y342F knock-in mice where the tyrosine at position 342 was mutated to phenylalanine. In this report, we demonstrate that phosphorylation of Y342 on Syk plays an important positive regulatory role in the signal transduction from an ITAM or a hemITAM receptor. We will also show that bleeding is unaffected in Syk Y342F mice while thrombus formation is significantly prolonged.

## Results

### Production and characterization of Syk Y342F knock-in mice

**Figure 1:**
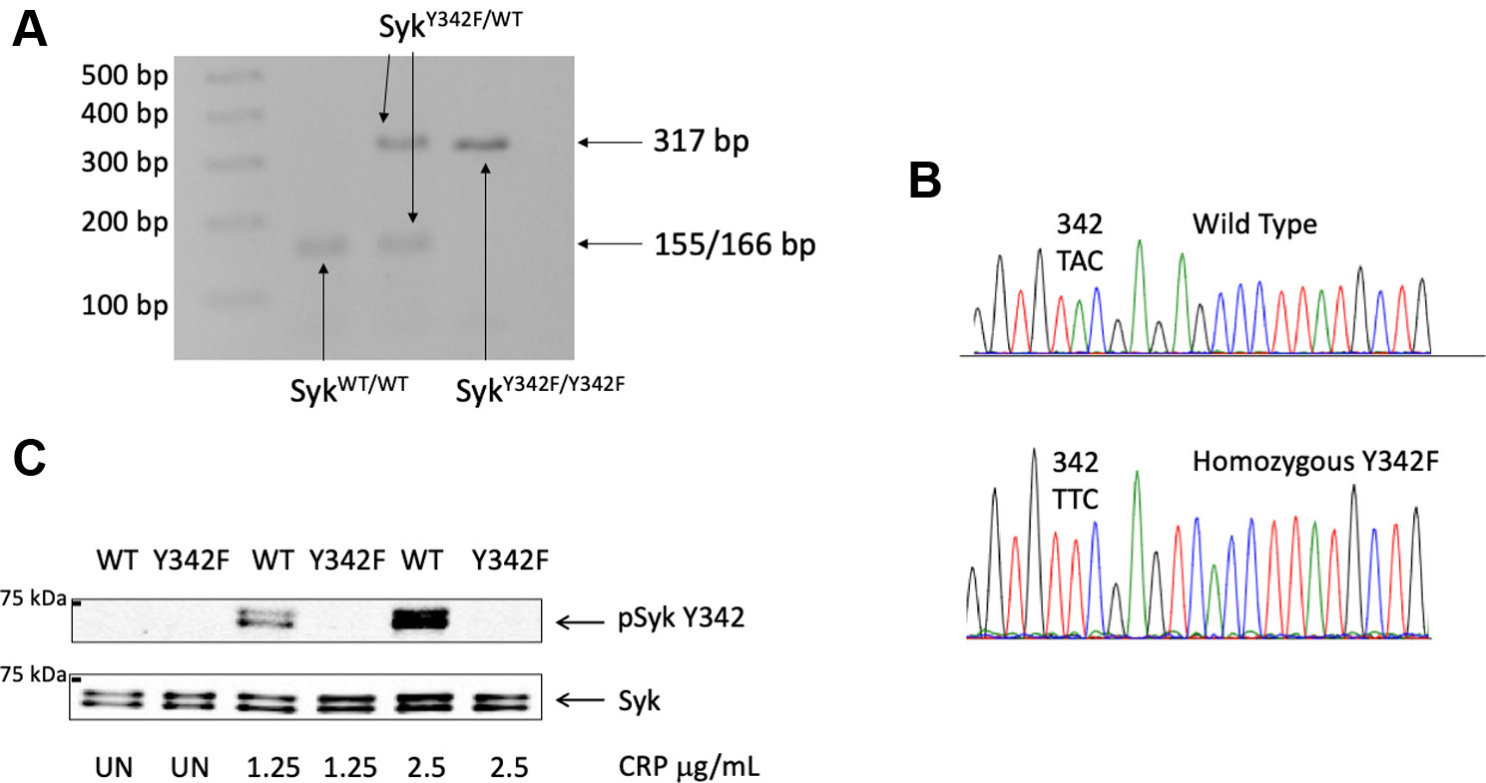
**Production of Syk Y342F knock-in mice.***A*, restriction enzyme digest of PCR products from Syk Y342F homozygous knock-in (Syk^Y342F/Y342F^), Syk Y342F heterozygous (Syk^Y342F/WT^), and WT littermate control (Syk^WT/WT^) DNA. Addition of Y342F eliminates the ability for RsaI to cleave the product leaving only the 317 bp band. *B*, sequence analysis of PCR products of WT and Y342F homozygous mouse DNA. *C*, representative Western blot showing that Syk Y342 phosphorylation is present in CRP-stimulated WT platelets but absent from Syk Y342F knock-in platelets. CRP, collagen-related peptide; Syk, spleen tyrosine kinase; UN, unstimulated sample.

Primary mast cells and basophilic cells in culture expressing Syk Y342F as a result of transfection or transduction demonstrated that tyrosine phosphorylation of mutated Syk and proteins important for ITAM-mediated signaling was reduced as compared with cells expressing WT Syk (40, 41). Therefore, to determine the function of Y342 in downstream signaling of hemITAM receptors and ITAM receptors, and phosphorylation of other sites on Syk, we produced an Syk Y342F knock-in mouse using the CRSPR/Cas9 technique through a commercial source (Cyagen). The Syk Y342F mutation was confirmed by both sequencing and digesting the PCR product with a restriction enzyme, RsaI. The oligonucleotides used for PCR are forward primer 5′-CTCCGCTGCATGCAACTGTC and reverse primer 5′-GCAGTGCAATGAGTCAACGGTGC. RsaI can cleave the WT nucleotide sequence (gtac) but cannot cleave the knock-in sequence (gttc). Therefore, the results after the digest are as follows: WT (+/+) 166 & 155 bp products, heterozygous (+/−) 155, 166, and 317 bp products, and homozygous (−/−) 317 bp product. An example of these PCR products is shown in Figure 1*A*. We also confirmed that the genotyping using sequencing of the PCR product (Fig. 1*B*). To confirm the Y342F mutation at the protein level, we isolated platelets from Syk Y342F and WT littermate control mice and stimulated them with collagen-related peptide (CRP). We performed Western blot analysis to visualize phosphorylation of Y342. We observed a robust band in WT mouse platelets treated with CRP at any concentration, but no band is present at the expected molecular weight in samples obtained from Syk Y342F mouse platelets (Fig. 1*C*). These data demonstrate that we successfully produced Syk Y342F knock-in mice.

### Syk Y342F knock-in mice are viable

**Figure 2:**
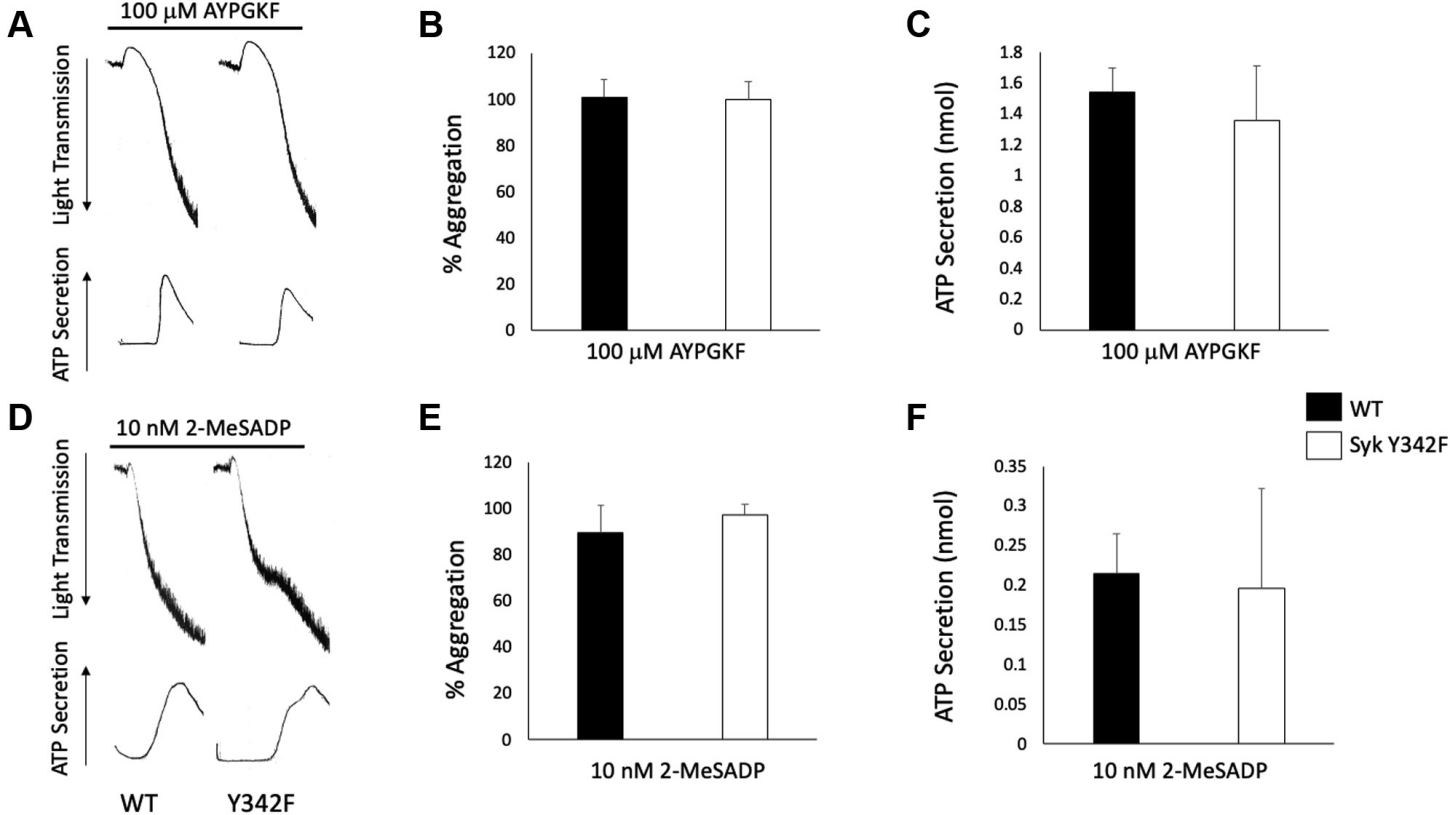
**GPCR-mediated platelet reactivity is intact in Syk Y342F mice.***A*, representative aggregation and secretion tracings of platelets from WT and Syk Y342F mice stimulated with the PAR4 agonist AYPGKF. *B*, quantitation of aggregation and *C*, secretion of platelets from Syk Y342F and WT littermate control mice stimulated with 100 μM AYPGKF. *D*, representative aggregation and secretion tracings of platelets from WT and Syk Y342F mice stimulated with the P2Y receptor agonist 2-MeSADP. *E*, quantitation of aggregation and *F*, secretion of platelets from Syk Y342F and WT mice stimulated with 10 nM 2-MeSADP. n = 5. GPCR, G protein–coupled receptor; Syk, spleen tyrosine kinase.

Heterozygous Syk Y342F knock-in mice and homozygous Syk Y342F knock-in mice bred normally and produced pups at expected Mendelian ratios. In contrast, Syk knockout mice are not viable and die perinatally (45, 46). Blood cell counts were not altered in Syk Y342F knock-in mice except for significantly reduced circulating red blood cells (Table 1). As all other cell counts were normal, we chose not to pursue the red blood cell number reduction during this study. However, it is an interesting finding and would be worth investigating later. Platelets from Syk Y342F mice respond normally to the G protein–coupled receptor agonists AYPGKF (PAR4) and 2-MeSADP (P2Y) (Fig. 2).

**Table 1:** Blood cell counts from Syk Y342F and WT littermate control mice

Genotype	WBC (K/ml)	NE (K/ml)	LY (K/ml)	RBC (M/ml)	PLT (K/ml)	MPV (fl)
Syk^WT/WT^	8.86 ± 0.80	0.92 ± 0.12	7.44 ± 0.71	9.76 ± 0.23	692.48 ± 20.79	4.07 ± 0.05
Syk^Y342F/Y342F^	10.54 ± 1.09	0.63 ± 0.13	9.22 ± 0.85	8.40 ± 0.13^a^	710.44 ± 66.02	3.98 ± 0.05

Abbreviations: LY, lymphocyte; MPV, mean platelet volume; NE, neutrophil; PLT, platelet; RBC, red blood cell; WBC, white blood cell.

a *p* < 0.05 compared with WT, n = 12.

### HemITAM-mediated aggregation and secretion is reduced in Syk Y342F platelets

**Figure 3:**
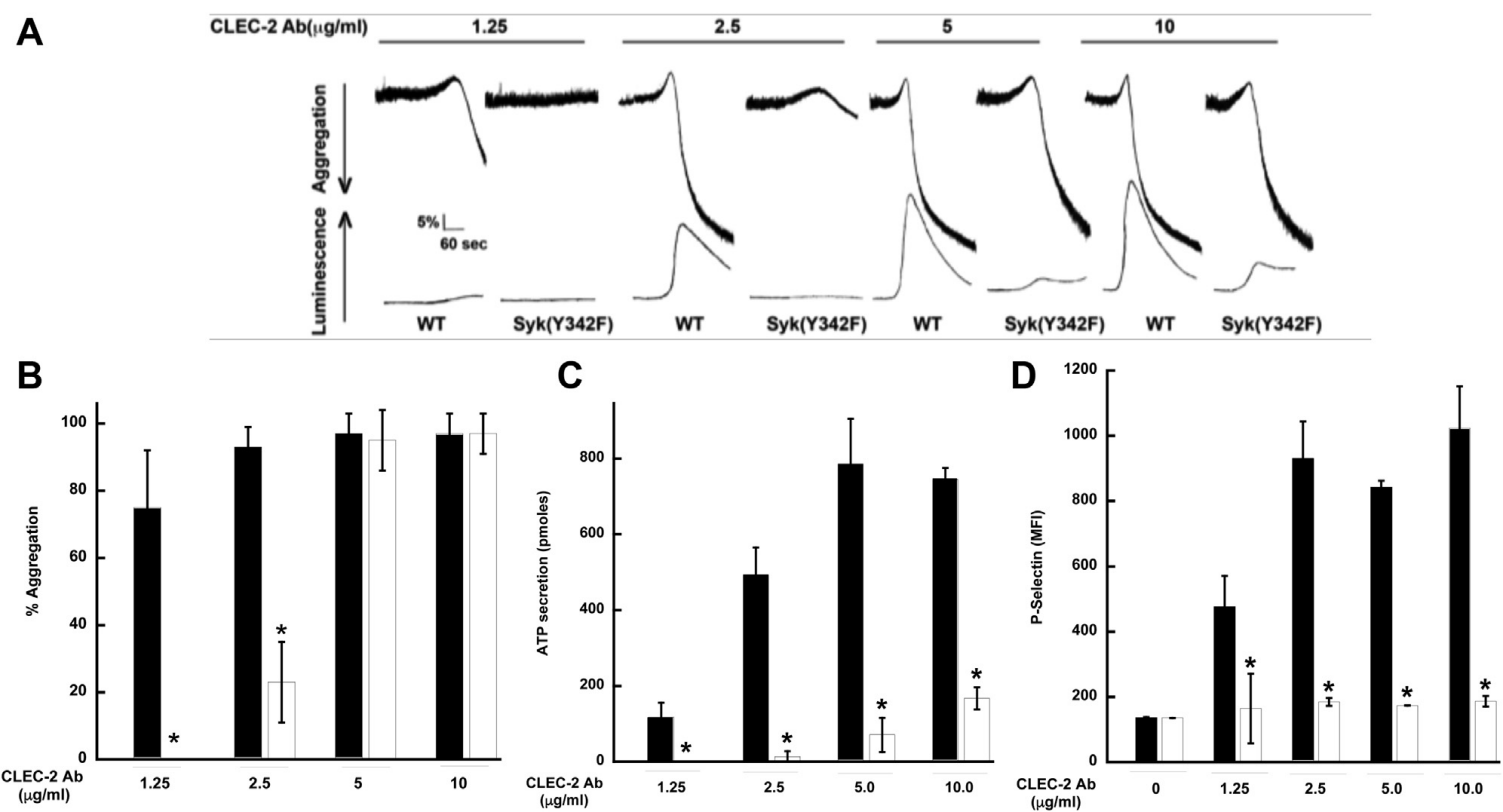
**Syk Y342F platelets react poorly to a CLEC-2 monoclonal antibody.***A*, aggregation and secretion were measured after the CLEC-2 receptor on WT or Syk Y342F platelets were stimulated with the indicated concentration of monoclonal CLEC-2 antibody. *B*, quantification of aggregation. *C*, ATP secretion. *D*, P-selectin surface expression from WT (*black bars*) or Syk Y342F (*white bars*) platelets after stimulation with CLEC-2 monoclonal antibody. ∗*p* < 0.05, n = 5. CLEC-2, C-type lectin-like receptor-2; Syk, spleen tyrosine kinase.

Syk is crucial for hemITAM-dependent signaling (12, 29, 33, 47). We wanted to investigate whether phosphorylation on Syk Y342 is essential for Syk activation and functions when CLEC-2 is activated. Therefore, we stimulated isolated platelets from Syk Y342F and WT littermate control mice with a monoclonal antibody that crosslinks the CLEC-2 receptor and monitored aggregation and ATP secretion. We were able to observe only minor aggregation or secretion in Syk Y342F platelets upon stimulation with a low concentration of CLEC-2 antibody, whereas there was robust aggregation and ATP secretion of WT control platelets (Fig. 3, *A*–*C*). This was not the case when a high concentration of CLEC-2 antibody was used as an agonist as full aggregation was observed in Y342F platelets although ATP secretion was still reduced (Fig. 3, *A*–*C*). We also evaluated alpha granule release using P-selectin as a marker by flow cytometry. As can be seen in Figure 3*D*, alpha granule release was also reduced in Y342F platelets compared with WT control platelets.

### HemITAM-mediated signaling is disrupted in Syk Y342F platelets

**Figure 4:**
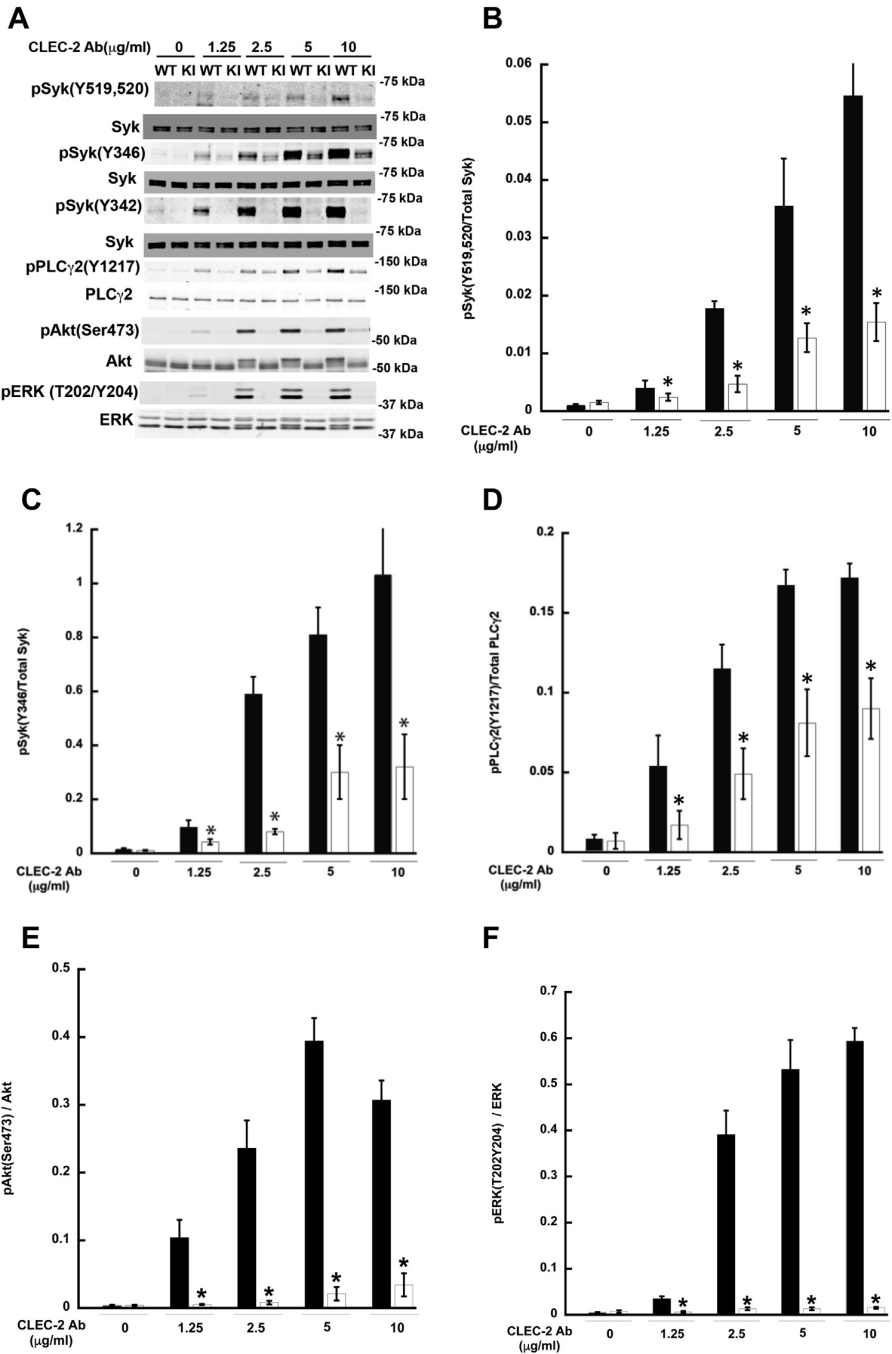
**HemITAM-mediated signaling is markedly reduced in Syk Y342F platelets.***A*, representative Western blots probed for the indicated total and phosphorylated proteins in WT littermate control (*black bars*) and Syk Y342F platelets (*white bars*) following CLEC-2 crosslinking. *B*–*F*, quantification of the phosphorylated to total protein ratios for the indicated protein following CLEC-2 crosslinking at the concentration indicated. ∗*p* < 0.05 compared with WT, n = 5. CLEC-2, C-type lectin-like receptor-2; ITAM, immune tyrosine activation motif; Syk, spleen tyrosine kinase.

As we demonstrated that Syk Y342 was necessary for platelet functional responses upon CLEC-2 clustering, we examined the phosphorylation status of Syk and PLCγ2 following stimulation of the CLEC-2 receptor in Syk Y342F and WT littermate control platelets. After treatment with the CLEC-2 antibody, there is phosphorylation of Syk Y519/520, Syk Y346, and PLCγ2 Y1217 in WT platelets, but phosphorylation of any of these sites is dramatically reduced in Syk Y342F platelets (Fig. 4, *A*–*D*). This suggests that the signaling required to enable platelet function is completely blocked with the loss of Y342 phosphorylation on Syk during low levels of CLEC-2 clustering. We further evaluated the role of Y342 phosphorylation on Akt and Erk pathways. As shown in Figure 4, *A*, *E* and *F*, both Akt and Erk phosphorylations were impaired in the Y342F platelets compared with WT control platelets.

### Syk Y342F platelets have impaired GPVI-mediated aggregation and secretion

**Figure 5:**
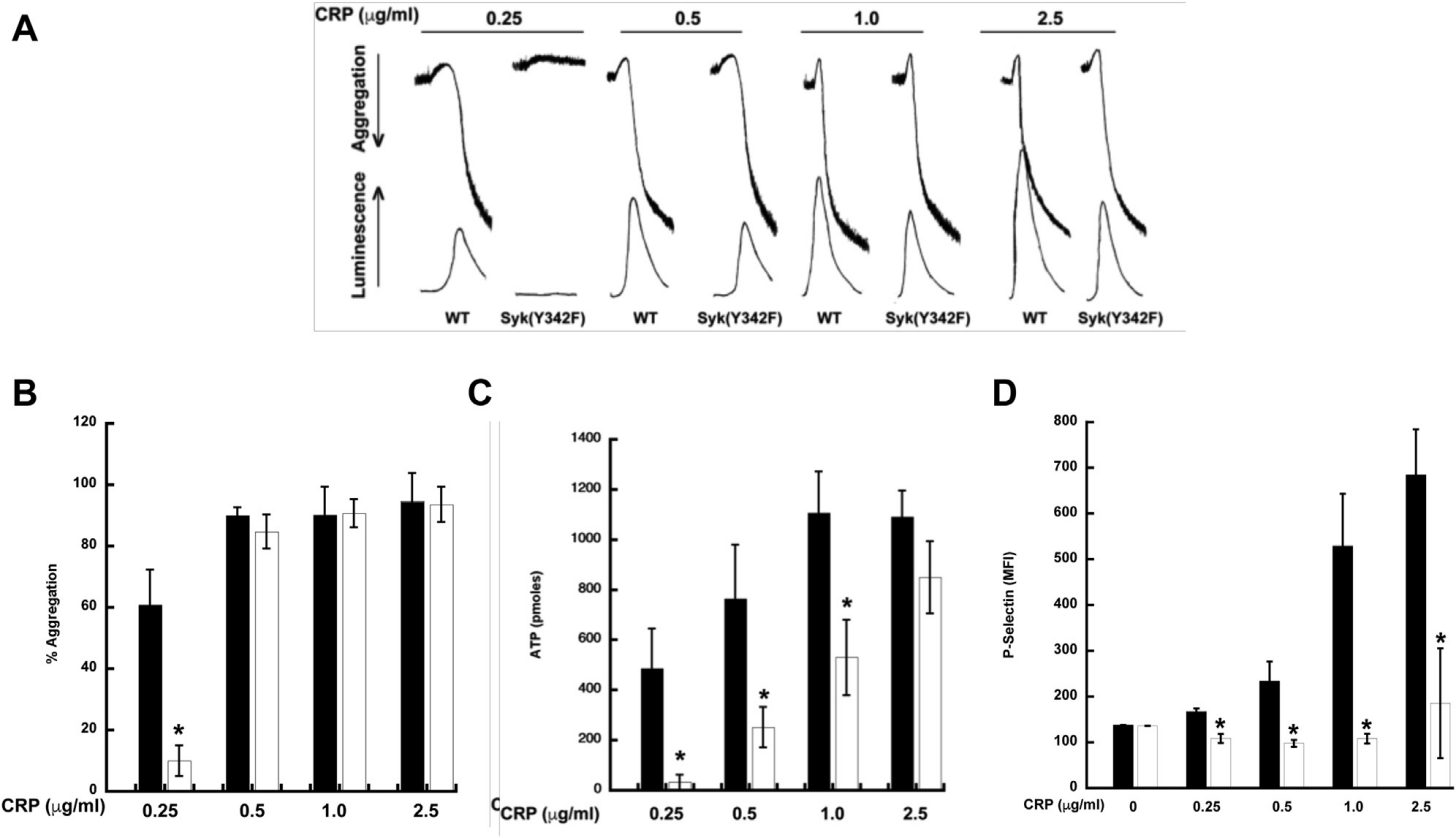
**Impaired GPVI-mediated aggregation and secretion in Syk Y342F platelets.***A*, representative aggregation and secretion tracings of Syk Y342F (*white bars*) and WT littermate control (*black bars*) platelets stimulated with the indicated concentrations of CRP. *B*, quantification aggregation. *C*, ATP secretion. *D*, P-selectin surface expression from multiple independent experiments. ∗*p* < 0.05 compared with WT, n = 6. CRP, collagen-related peptide; GPVI, glycoprotein VI; Syk, spleen tyrosine kinase.

Because Syk activity is vital to platelet activation *via* GPVI agonists just as it is with CLEC-2 agonists, we evaluated the effect of Syk Y342F on GPVI-mediated aggregation and secretion, using isolated platelets from Syk Y342F and WT littermate control mice with varying concentrations of CRP. We observed pronounced aggregation and secretion in WT platelets (Fig. 5*A*). However, Syk Y342F platelets had significantly less aggregation and secretion at the lower concentrations of CRP, this defect was not observed when a high concentration of CRP was used (Fig. 5, *A*–*C*). As can be seen in Figure 5*D*, alpha granule release was also reduced in Y342F platelets compared with WT control platelets.

### GPVI-mediated signaling is perturbed in Syk Y342F platelets

**Figure 6:**
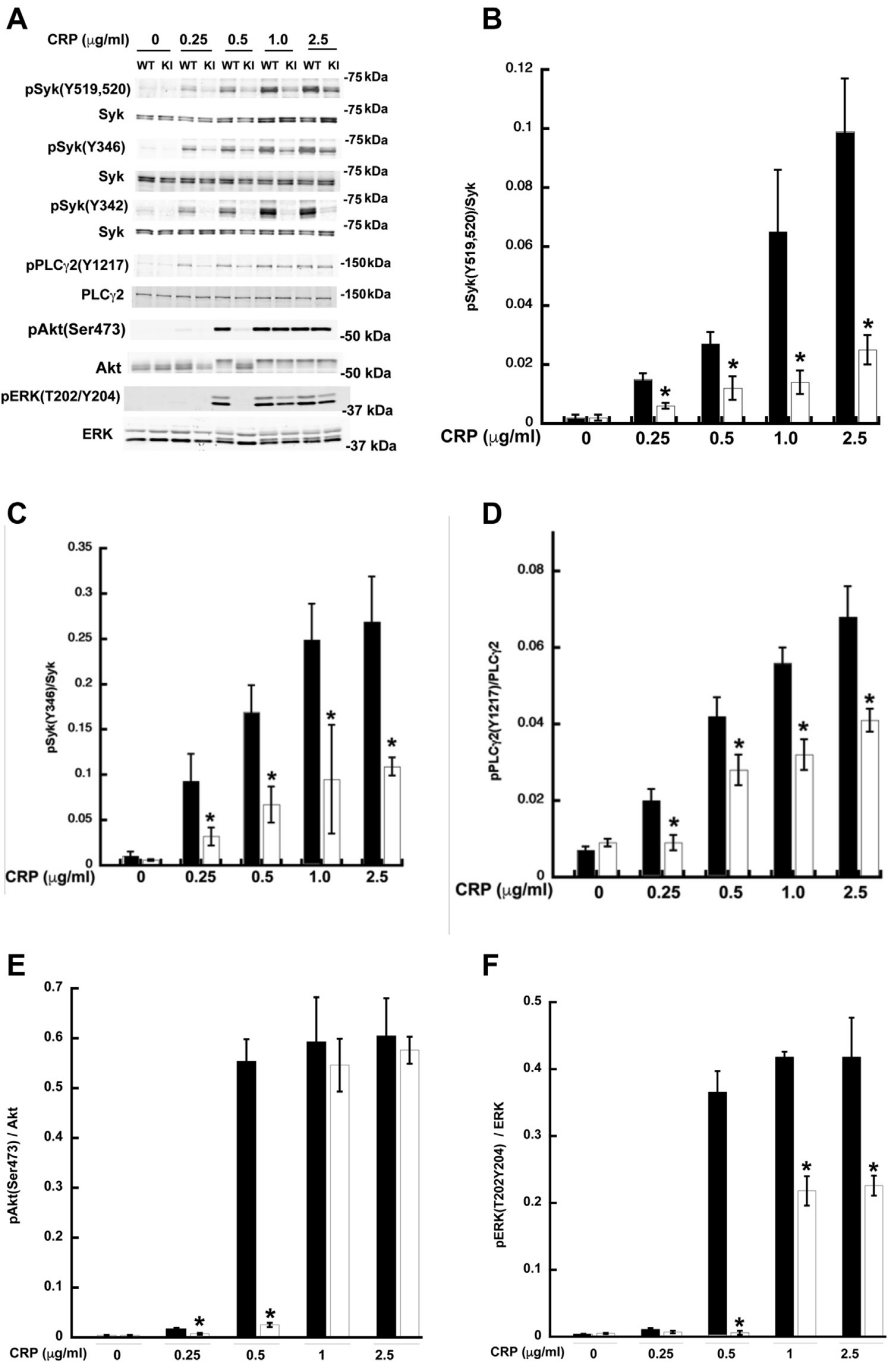
**ITAM-mediated signaling is hindered in Syk Y342F platelets.***A*, representative Western blots showing the indicated phosphorylated and total protein in Syk Y342F (*white bars*) and WT littermate control (*black bars*) platelets stimulated with various concentrations of CRP for 3 min. *B*–*F*, quantitation of the indicated phosphorylated to total protein ratio for the indicated time point. ∗*p* < 0.05 compared with WT, n = 6. CRP, collagen-related peptide; ITAM, immune tyrosine activation motif; Syk, spleen tyrosine kinase.

Syk is a vital component of the signaling pathway downstream from hemITAM- as well as ITAM-bearing receptors (21, 22, 23, 24). Therefore, we analyzed several proteins involved within the signaling cascade downstream of GPVI stimulation. After activation with CRP, there is phosphorylation of Syk Y519/520, Syk Y346, and PLCγ2 Y1217, in WT platelets, but phosphorylation of any of these sites is dramatically reduced in Syk Y342F platelets (Fig. 6, *A*–*D*). We further evaluated the role of Y342 phosphorylation on Akt and Erk pathways. As shown in Figure 6, *A*, *E* and *F*, both Akt and Erk phosphorylations were impaired in the Y342F platelets compared with WT control platelets. These data collectively indicate that signaling downstream of GPVI is diminished in Syk Y342F platelets.

### Intracellular calcium mobilization is disrupted in Syk Y342F platelets

**Figure 7:**
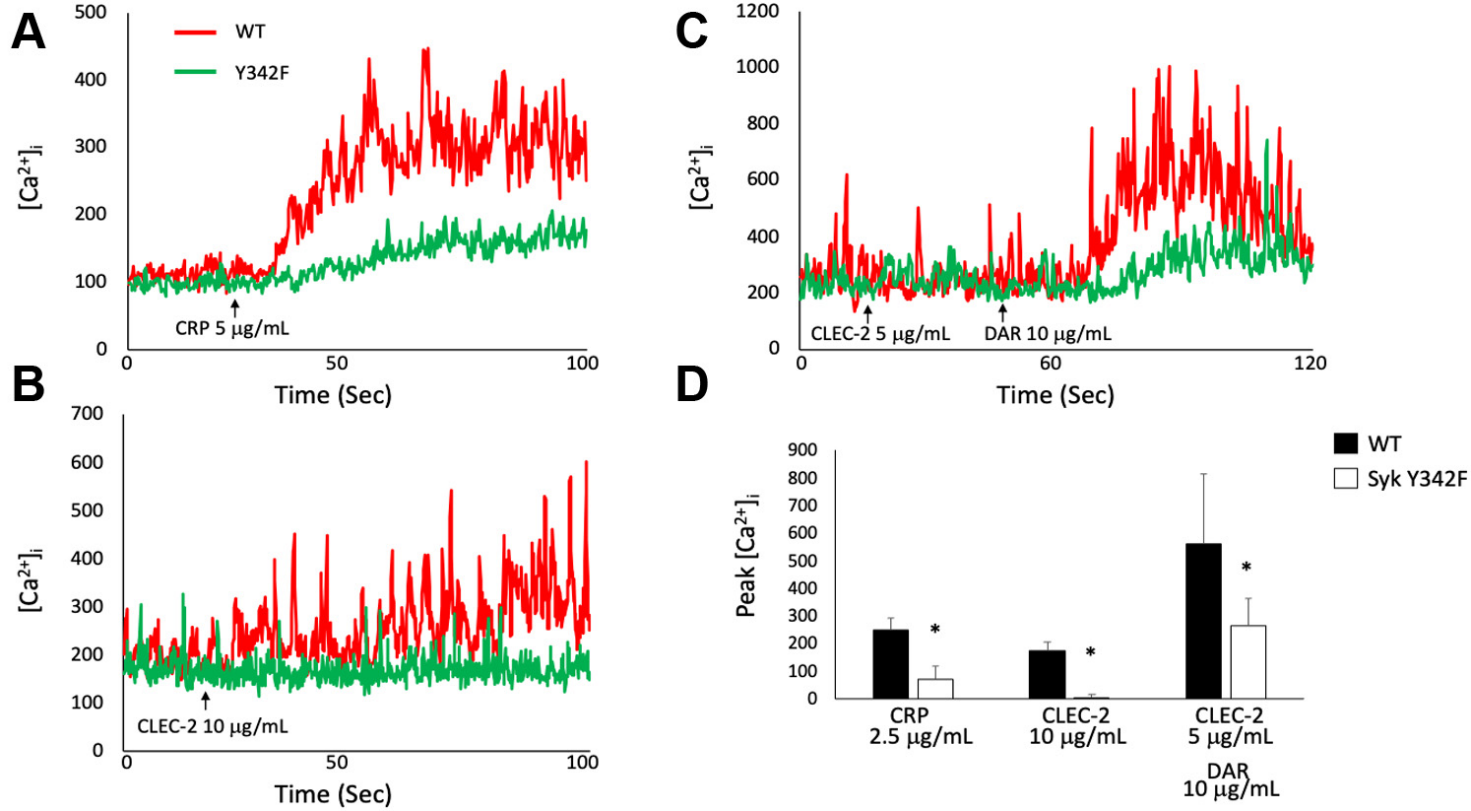
**Calcium mobilization is greatly impaired in Syk Y342F platelets.***A*–*C*, representative tracings from measurements of calcium mobilization in response to the indicated agonist over time. *D*, quantitation of the maximum calcium concentration recorded following stimulation with the indicated agonist minus the baseline (*background*) reading, which was taken 30 s prior to the addition of agonist. ∗*p* < 0.05 compared with WT, n = 5. Syk, spleen tyrosine kinase.

A hallmark of platelet activation is the mobilization of intracellular calcium. Therefore, we measured calcium flux in Syk Y342F and WT littermate control platelets after stimulation with different agonists. In response to CRP, we observed a robust calcium signal in WT platelets (Fig. 7*A*). However, calcium mobilization in Syk Y342F platelets was significantly reduced compared with WT platelets (Fig. 7*D*). Using the CLEC-2 monoclonal antibody, we detected a weak but measurable signal from WT platelets, but no signal was detectable in the Syk Y342F platelets (Fig. 7, *B* and *D*). CLEC-2 double crosslinking produced the maximum calcium response in WT and Syk Y342F platelets though calcium mobilization was still significantly reduced in Syk Y342F platelets (Fig. 7, *C* and *D*). Collectively, these data demonstrate that phosphorylation of Y342 on Syk is important for calcium mobilization in response to both ITAM- and hemITAM-mediated activation. Although these data agree with the notion that platelet functional responses can be reduced by Syk Y342F, there are quantitative differences between functional and calcium flux data; Syk Y342F reduces calcium responses even under conditions of strong stimulation, when it exerts no significant effect on aggregation.

### Syk Y342 is important for *in vivo* thrombus formation but dispensable for hemostasis

**Figure 8:**
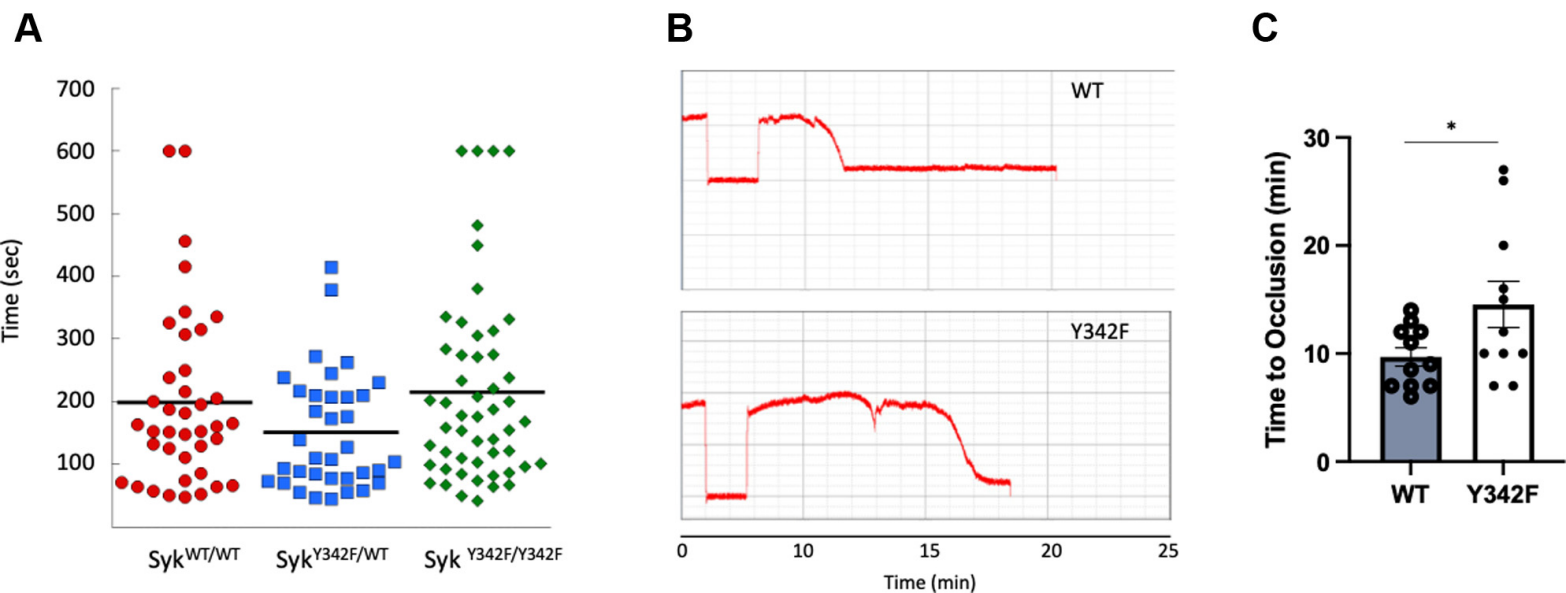
***In vivo* thrombus formation is perturbed in Syk Y342F knock-in mice.***A*, scatter plot showing the time it took for bleeding to stop during tail bleeding experiments conducted on homozygous Syk^Y342F/Y342F^ knock-in, heterozygous Syk^Y342F/WT^, and WT (Syk^WT/WT^) littermate control mice 4 to 6 weeks of age in a blind fashion. *B*, representative tracings of blood flow through the carotid artery in one WT littermate control and one Syk Y342F knock-in mouse following the application of 7.5% FeCl_3_ for 90 s. *C*, quantitation of the time to occlusion in WT and Syk Y342F knock-in mice following 7.5% FeCl_3_ injury on the carotid artery. ∗*p* < 0.05 compared with WT, n = 11. Syk, spleen tyrosine kinase.

To determine the impact of Syk Y342F on hemostasis, we performed tail bleeding assay on litter-matched WT, heterozygous, and homozygous Syk Y342F mice. We found no differences in the bleeding times (Fig. 8*A*). These data suggest that Syk Y342 phosphorylation is not essential for hemostasis. To determine whether thrombus formation is impacted by the impaired phosphorylation of Y342 on Syk, we injured the carotid arteries of Syk Y342F and WT littermate control mice with 7.5% FeCl_3_ for 90 s and monitored occlusion time. WT mice formed stable occlusions in just under 10 min on average, whereas Syk Y342F mice took significantly longer to form stable occlusions (Fig. 8, *B* and *C*). These data suggest that phosphorylation of Y342 on Syk positively regulates thrombus formation *in vivo*.

## Discussion

Syk is phosphorylated on many tyrosine residues, which appear to interact in complex ways. Hence, dissecting the role of each individual tyrosine residue will provide crucial insight into Syk activation mechanisms. Previously, we demonstrated that downstream of CLEC-2, Tec kinases are critical for Syk phosphorylation (29). The current models of Syk activation postulate a key role of Syk binding to either phosphorylated ITAM or hemITAM and its subsequent phosphorylation in this process. Previous reports indicated that Syk Y342 and other tyrosines in the linker region exert a substantial regulatory effect on Syk activation and signaling, and the role of Y342 is crucial (8, 39, 40, 41, 42, 43). However, whether Y342 phosphorylation is crucial for signaling and activation *via* hemITAM receptors is not known. Focusing our effort of the Y342 site, we clearly demonstrated that platelet functional responses induced by CLEC-2 signaling were greatly reduced in Syk Y342F knock-in mice compared with WT littermates. Consistent with this result, phosphorylation of Syk Y519/520 and PLCγ2 Y1217 and calcium responses were also greatly reduced. Syk Y346 phosphorylation was likewise greatly reduced by Syk Y342F; this result is consistent with the effect of Y342F on phosphorylation of Y346 that we have demonstrated previously in the reconstituted cell system transfected to express Syk Y342F (8). These data are in support of the critical role of Syk Y342 in the tyrosine phosphorylation–mediated activation of Syk, perhaps that of a primary target of kinases activating Syk. A counter point of view to this explanation is that phosphorylation of another tyrosine residue on Syk results in autophosphorylation of Y342. At this time, we cannot test this possibility without generating additional specific tyrosine knock-in mice.

Our data support the notion that Y342 is a crucial regulator of Syk activity upon GPVI stimulation, as phosphorylation of Y519/520 is greatly reduced in Syk Y342F platelets. However, the very fact of Y519/520 phosphorylation in the absence of pY342 suggests that Y519/520 may be phosphorylated upon GPVI stimulation independently of Y342 phosphorylation. A rather close correlation of the phosphorylation levels for Y519/520 and Y346 suggests that this regulatory effect may be complex and involve several tyrosine sites. Furthermore, even though there is far less Y519/520 phosphorylation in Syk Y342F platelets compared with WT platelets, there is still robust platelet functional responses when higher concentrations of agonists, especially CRP, are used. These data strongly suggest that suboptimal Syk Y519/520 phosphorylation is sufficient to provide enough active Syk to transduce an adequate signal. Thus, the aggregatory and secretory responses in Syk Y342F platelets after stimulation with 5 μg/ml CRP are nearly identical to that of WT platelets, although Syk Y519/520 phosphorylation is greatly reduced compared with WT control. We cannot rule out that Y519/520 phosphorylation may only be consequential autophosphorylation and that Syk activation is independent of Y519/520 phosphorylation. In agreement with this notion, it was shown previously using BaF3 cells that Syk is capable of transmitting a downstream signal even when Y525 and Y526 (human Syk residues homologous to mouse Y519 and Y520) are mutated to phenylalanine (48). Furthermore, when examining Syk expressed in COS cells, it was revealed that the double mutant Syk YY518/519FF (porcine Syk residues homologous to mouse Y519 and Y520) retained about 60% of its activity in an *in vitro* kinase assay (35). In the same cell line, when Syk is double mutated as aforementioned, cellular tyrosine phosphorylation is greatly reduced unless the SFK, Lck is coexpressed, in which case tyrosine phosphorylation is almost completely restored (49). These data suggest that while phosphorylation of Y519/520 (or similar sites conserved among species) located in the activation loop of Syk may be used as a marker of Syk activity, it may not be essential for this activity. Furthermore, there may be another phosphorylation site on Syk that is more indicative of Syk activity, and we are currently pursuing this line of investigation. The importance of Y519/520 phosphorylation is currently under investigation through the generation of additional knock-in mice.

Syk Y342 phosphorylation is crucial to transduction of a weak signal downstream of GPVI, but it may not be essential for transmitting stronger signals. Our data show that Syk Y342F platelets have a greatly diminished response to low concentrations of CRP (Fig. 5). However, platelet functional response was comparatively normal when Syk Y342F platelets were stimulated with high concentrations of CRP. Hence, upon stronger signal from GPVI, there might be other phosphorylations through other SFK members that obliviate the need for Y342 phosphorylation.

Syk Y342F mice have reduced thrombus formation in a ferric chloride injury model but do not have a bleeding phenotype. This is likely linked to dense granule secretion. It has been postulated that secretion defects, which manifest at low agonist concentrations, but recover at high agonist doses (like Syk Y342F mouse platelets treated with CRP), are more likely to correlate with alterations in thrombosis than alterations in hemostasis (50). These data suggest that Syk Y342 phosphorylation is important for thrombus formation consistent with reduced platelet functional responses in case of collagen or low CRP stimulation in Y342F knock-in mice.

In conclusion, we demonstrate that Y342 on Syk positively regulates ITAM- and hemITAM-mediated signaling using an Syk Y342F knock-in mouse model, specifically at low agonist concentrations. Physiologically, Syk Y342 appears to positively regulate thrombus formation, without affecting bleeding times.

## Experimental procedures

### Antibodies and reagents

All reagents were purchased from Thermo Fisher Scientific unless otherwise stated. Collagen and chronolume, used for the detection of secreted ATP, were purchased from Chrono-Log Corporation. The CLEC-2 activating antibody was purchased from BioLegend, and the donkey antirat immunoglobulin was purchased from Novex. Anti-pSyk Y525/526 (mouse Y519/520) and anti-pPLCγ2 (Y1217) were purchased from Cell Signaling Technology. Anti-pSyk Y352 (Y346 in mouse) and anti-pSyk Y348 (Y342 in mice) were purchased from Abcam. Anti-Syk and anti-PLCγ2 were purchased from Santa Cruz Biotechnology. Ibrutinib was purchased from Selleckchem. Odyssey blocking buffer and secondary antibodies IRDye 800CW goat anti-rabbit and IRDye 680LT goat antimouse were purchased from Li-Cor. CRP-XL was purchased from Dr Richard Farndale at the University of Cambridge. AYPGKF was purchased from GenScript.

### Animal housing and production

Mice were housed in a pathogen-free facility, and all animal procedures were approved by the Temple University Institutional Animal Care and Use Committee (protocol #4864). Syk Y342F mice were produced by Cyagen on a fee-for-service basis.

### Preparation of mouse platelets

Mouse blood was collected, and platelets were isolated as previously described (51). The resulting platelets were counted using a Hemavet 950FS blood cell analyzer (Drew Scientific). Platelet counts were adjusted to a final concentration of 1.5 × 10^8^ cells/ml in *N*-2-hydroxyethylpiperazine–*N*′-2-ethanesulfonic acid–buffered (pH 7.4) Tyrode’s solution containing 0.2 U/ml apyrase.

### Platelet aggregation and ATP secretion

All platelet aggregation and secretion experiments were carried out using a Lumi-Aggregometer (Chrono-Log) at 37 °C under stirring conditions. Platelet aggregation was measured using light transmission, and ATP secretion was measured using Chrono-Lume (a luciferin/luciferase reagent).

### Western blotting

Western blotting procedures were performed as described previously (51). Briefly, platelets were stimulated for the indicated time points in a Lumi-Aggregometer with either a GPVI or a CLEC-2 agonist. The reaction was stopped by precipitating the platelet proteins using 0.6 N HClO_4_ and washed with water prior to the addition of sample loading buffer. Platelet protein samples were then boiled for 5 min prior to resolution by SDS-PAGE and transferred to nitrocellulose membranes. The membranes were then blocked using Odyssey blocking buffer and incubated overnight with primary antibodies against the indicated protein. The membranes were then washed with Tris-buffered saline containing 0.1% Tween-20 prior to incubation with appropriate secondary antibodies for 1 h at room temperature. The membranes were washed again and imaged using a Li-Cor Odyssey infrared imaging system.

### Flow cytometry

Surface exposure of P-selectin (antiFITC-P-selectin) in murine platelets was determined as previously described (52). The samples were analyzed using a FACSCalibur flow cytometer (BD Biosciences). Appropriate isotype controls were used.

### Intracellular calcium mobilization

Measurement of intracellular calcium was conducted as described previously with minor modifications (53). Whole blood drawn from mice was anticoagulated with 3.8% sodium citrate and diluted with four volumes of Pipes acid–buffered Tyrode’s buffer (pH 6.5) containing 1 μM PGE1 and 0.2 U/ml apyrase. The samples were then centrifuged at 100*g* for 10 min. The resulting platelet-rich plasma was collected and diluted with two volumes in the aforementioned Pipes Tyrode’s buffer. Platelets were then centrifuged at 800*g* for 10 min at room temperature, and the resulting pellet resuspended in 1 ml Pipes Tyrode’s buffer and incubated with 5 μM FURA-2 AM for 45 min at room temperature. The samples were then diluted with 1 ml Pipes Tyrode’s buffer and centrifuged at 400*g* for 10 min. The platelet pellet was then resuspended with *N*-2-hydroxyethylpiperazine–*N*′-2-ethanesulfonic acid–buffered Tyrode’s buffer, adjusted to 1.5 × 10^8^ ml, and allowed to rest for 30 min prior to calcium measurements. Calcium was measured using a Duetta fluorescence and absorbance spectrometer from Horiba using the following equation: [Ca^2+^]_i_ = (*K*_*d*_(f-fmin)/(fmax-f)).

### Tail bleeding assay

Mouse tail bleeding was conducted as previously described (54). Mice aged 4 to 6 weeks were anesthetized prior to amputation of the distal 3 mm of the tail. The tail was then immersed in 37 °C saline, and bleeding was monitored. If bleeding continued for greater than 600 s, then bleeding was halted manually by applying pressure.

### Carotid artery injury

FeCl_3_ was used to injure the carotid artery as previously described (54). Mice aged 10 to 12 weeks were anesthetized, and the carotid artery was exposed. A baseline blood flow reading was obtained using a Transonic T402 flow meter. The carotid artery was injured using a 1 × 1 mm piece of filter paper saturated with 7.5% FeCl_3_ for 90 s. The filter paper was removed, and blood flow was recorded.

### Statistics

All statistical analyses were performed using Microsoft Excel, and data were analyzed using a Student’s *t* test where *p* < 0.05 was considered statistically significant. All the data are presented as means ± SD of at least three independent experiments.

## Data availability

All data concerning this report are available in the article.

## Conflict of interest

The authors declare that they have no conflicts of interest with the contents of this article.
